# Pulmonary vein aneurysm in a New Zealand White rabbit: a case report

**DOI:** 10.1186/s42826-022-00114-7

**Published:** 2022-02-16

**Authors:** Jong-Min Kim, Chang Gok Woo, Sa Hun Kim, Eui-Suk Jeong, Kil-Soo Kim, Byeong-Cheol Kang

**Affiliations:** 1 Department of Experimental Animal Research, Biomedical Research Institute, Seoul National University Hospital, Seoul, 110-799 Korea; 2 Department of Pathology, Chungbuk National University College of Medicine, Cheongju, 361-763 Korea; 3 Laboratory Animal Center, Daegu-Gyeongbuk Medical Innovation Foundation, Daegu, Republic of Korea; 4 Graduate School of Translational Medicine, Seoul National University College of Medicine, 101 Daehakro, Jongno-gu, Seoul, 03080 Korea; 5 Biomedical Center for Animal Resource and Development, Seoul National University, College of Medicine, Seoul, Korea; 6 Designed Animal and Transplantation Research Institute, Institute of Green Bio Science Technology, Seoul National University, Pyeongchang-gun, Gangwon-do, Korea

**Keywords:** Pulmonary vein aneurysm, NZW rabbit, Spontaneous rupture

## Abstract

**Background:**

Pulmonary venous aneurysm (PVA) is a rare condition characterized by aneurysmal dilatation of the pulmonary vein in humans. The diagnosis is incidental usually as there are no clinical symptoms. This case report describes a histological diagnosis of PVA in a New Zealand White rabbit.

**Case presentation:**

A 1.5-kg male New Zealand White rabbit was acclimatized in an animal room for 5 weeks until the experiment began. However, the rabbit was found dead, with signs of nasal hemorrhage. Necropsy revealed tracheal and pulmonary hemorrhage, and the epistaxis had a pulmonary origin. PCR and ELISA to detect antigens and antibodies pertaining to the rabbit hemorrhagic disease virus showed negative results. Multiple ballooning lesions (50–200 μm size) in the pulmonary veins were observed on histological examination, and PVA was diagnosed. Death was attributed to a spontaneous rupture of the PVA and massive hemorrhage into the lung parenchyma that extended into the trachea and nasal passages.

**Conclusions:**

To the author’s best knowledge, this is the first report of a PVA in a rabbit.

## Background

In 1991, the New Zealand White (NZW) rabbit was procured from Kitayama Labs K.K. in Nagano Prefecture, Japan by Charles River Canada, which enforces stringent biosecurity practices for breeding its NZW rabbits to ensure the highest standards in health and genetics. Worldwide, the NZW rabbits are maintained as an outbred colony and are widely used for biomedical research in pharmacology, toxicology, teratology, and antibody production because of their small size, relatively low maintenance cost, low husbandry effort, ease of handling for routine clinical procedures, rapid reproductive turnover, and a lower incidence of zoonoses than other large animals. Pulmonary venous aneurysm (PVA) is a rare condition that is characterized by an aneurysmal dilatation of the pulmonary vein in humans [1]. Clinical symptoms are usually absent, and the diagnosis is incidental [1]. We report a case of histologically confirmed PVA in a NZW rabbit that apparently died of nasal hemorrhage.

## Case presentation

**Fig. 1:**
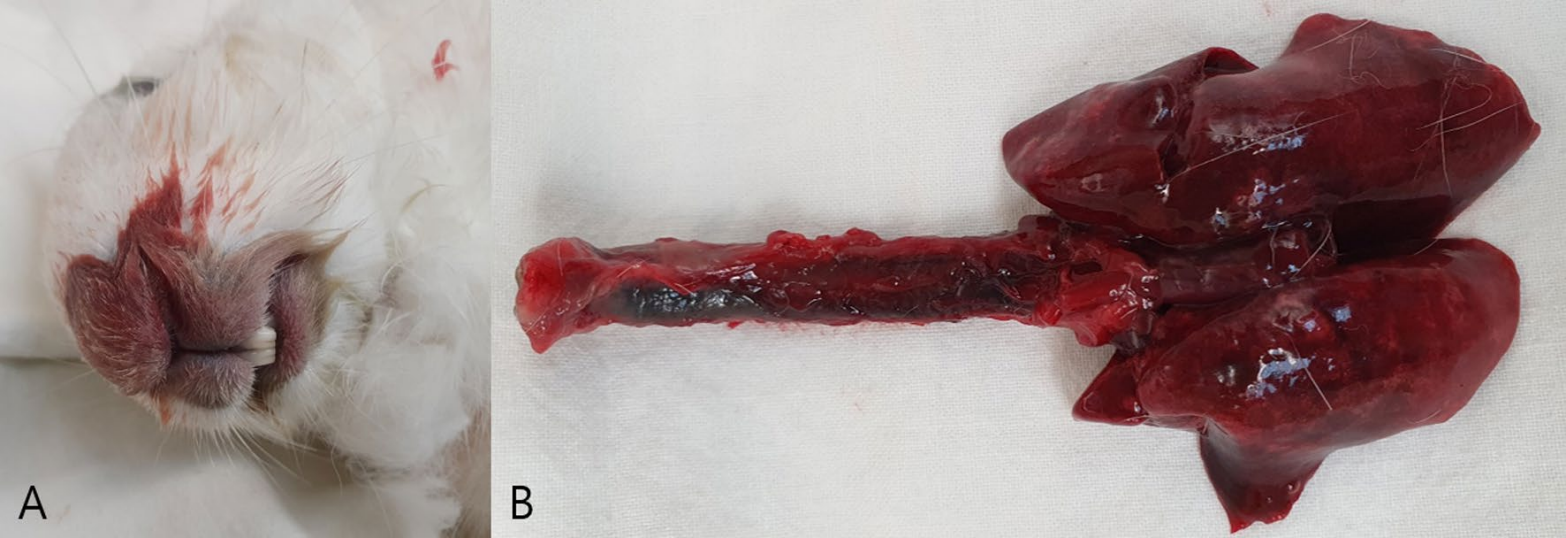
Nasal hemorrhage (**a**) and hemorrhage into the trachea and lung (**b**) in an NZW rabbit

**Fig. 2:**
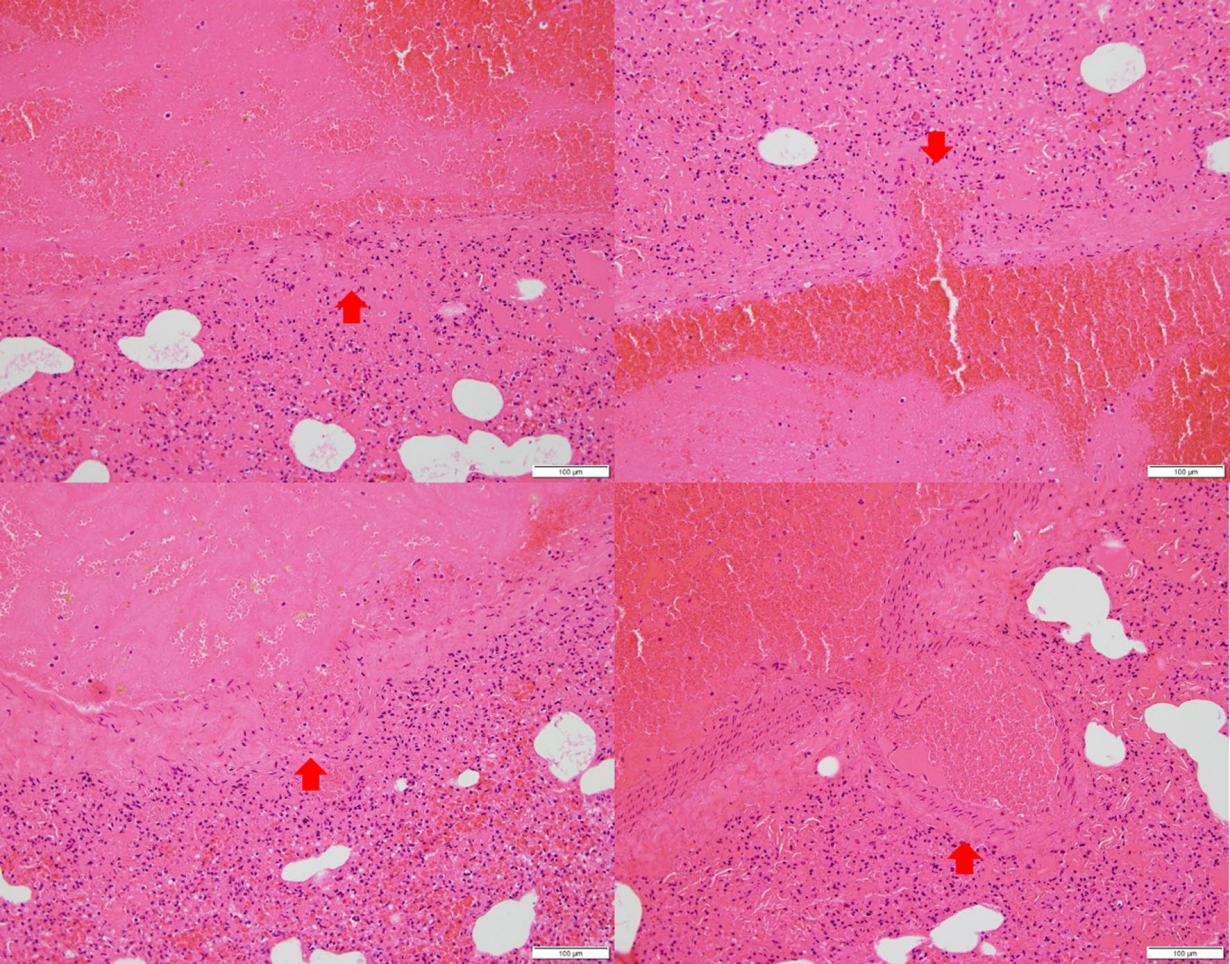
Histological images of aneurysms of pulmonary vein. Ballooning and thinning of vessel wall in pulmonary veins are indicated (arrow). Large deposits of red blood cells, fibrin, and lymphocytes in lung parenchyma. Bar = 100 μm

**Fig. 3:**
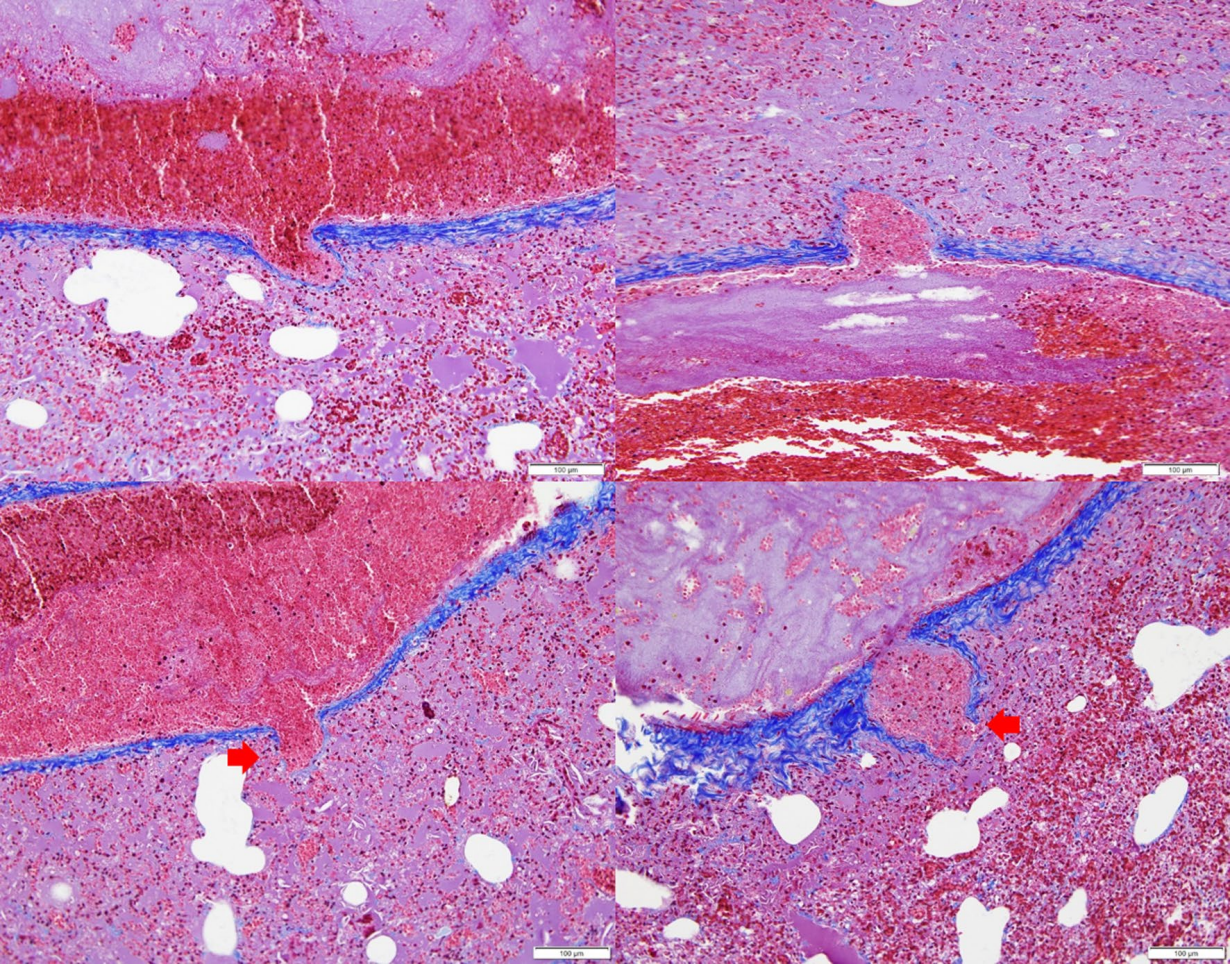
The presence of an aneurysmal and thin-walled vascular wall is confirmed on Masson Trichrome staining. A focal rupture of the wall is identified in the aneurysmal tip (arrow). Bar = 100 μm

Animal experiments were approved by the Institutional Animal Care and Use Committee (IACUC) of the Biomedical Research Institute at the Seoul National University Hospital (an Association for Assessment and Accreditation of Laboratory Animal Care-accredited facility; IACUC number: 18-0150). A 1.5-kg male NZW rabbit was acclimatized for 5 weeks in an animal room until the experiment commenced. The rabbit was found dead with apparent epistaxis (Fig. 1a). Necropsy revealed tracheal and pulmonary hemorrhages (Fig. 1b), and the epistaxis was confirmed to have originated from the lung. PCR testing and ELISA for the antigen and antibody of the rabbit hemorrhagic disease virus were negative. Histopathological examination revealed multiple ballooning lesions (size 50–200 μm) of the pulmonary veins with large fibrin and WBC deposits in the lung parenchyma (Fig. 2). A focal rupture of the venous wall was identified in the aneurysmal tip (Fig. 3), and a PVA was diagnosed. Death was attributed to a spontaneous PVA rupture followed by massive hemorrhage into the lung parenchyma that extended into the trachea and nasal passages.

## Discussion and conclusions

A literature search revealed two case reports of multiple endometrial venous aneurysms with acute-onset hemorrhagic vulvar discharge in pet rabbits (Oryctolagus cuniculus) [2, 3]. A case series reported endometrial venous aneurysms in 3 NZW laboratory rabbits [4]. In the present case, a PVA was diagnosed through histopathology. Pulmonary veins maintain a blood pressure of 4–15 mmHg, which is higher than that of uterine veins, which suggests a higher probability of hemorrhage with a PVA than with an endometrial venous aneurysm. In humans, aneurysms of the endometrial, vaginal, and cervical blood vessels are considered congenital [4]. An aneurysms in the genital tract can rupture and bleed as a complication of increased pressures during pregnancy or trauma [4]. In NZW rabbits, endometrial venous aneurysms are considered congenital, as there is no evidence of other predisposing pathological factors [4]. A congenital etiology is consistent with findings in the present case as the rabbit had no prior medical problems or any history of trauma. A limitation of this case report is that, unfortunately, we could not confirm the presence of venous aneurysms in the entire organ because the entire organ tissue was not preserved following necropsy. All venous aneurysms in rabbits that have been reported so far have been endometrial; however, in the present case, the subject was male; therefore, it is regrettable that venous aneurysms in the genital mucosa were not detected due to the unavailability of the male genital organs/tissues. However, we checked but found no venous aneurysms in tracheal tissue, which was the only other internal organ that was available. An in-depth study of venous aneurysms in rabbits is needed.

Aneurysms can occur when the structure or function of the connective tissue within the blood vessels is damaged, thereby weakening the vessel wall [5]. Aneurysms indicate that the intrinsic quality of the connective tissue in the vessel wall is poor (e.g., congenital defects and inflammation), and proteases may further alter the extracellular matrix and smooth muscle fibers, as in cases of acquired aneurysms [5]. In humans, syndromes characterized by defective synthesis of elastin or collagens I and III, vitamin C deficiency, atherosclerosis, systemic hypertension, trauma, vasculitis, congenital defects, and infection can result in aneurysms [5]. Although PVA is rare in humans, if PVA is present in several rabbits, they could constitute an animal model of PVA for research applications. Thus, further study using micro-CT of PVA in NZW rabbits is needed.

In conclusion, we described the diagnosis of PVA in an NZW rabbit. There are three case reports of endometrial venous aneurysms in rabbits; however, this is the first case report of a PVA in an NZW rabbit and indicates the possibility of a congenital defect in the venous vasculature of rabbits.

## Data Availability

Not applicable.

## Abbreviations

AAALAC: Association for Assessment and Accreditation of Laboratory Animal Care
IACUC: Institutional Animal Care and Use Committee
PVA: Pulmonary vein aneurysm
WBC: White blood cell
NZW: New Zealand White
